# A bibliometric analysis of clinical research on fracture-related infection

**DOI:** 10.1155/2022/8171831

**Published:** 2022-04-14

**Authors:** Cheng Li, Andrew L. Foster, Nicholas Hang Bao Han, Andrej Trampuz, Michael Schuetz

**Affiliations:** ^1^Beijing Jishuitan Hospital, Fourth Clinical College of Peking University, Department of Adult Joint Reconstructive Surgery, Beijing, China; ^2^Charité-Universitätsmedizin Berlin, Corporate Member of Freie Universität Berlin, Humboldt-Universität Zu Berlin, And Berlin Institute of Health, Center for Musculoskeletal Surgery (CMSC), Berlin, Germany; ^3^Department of Orthopaedic Surgery, Royal Brisbane and Women's Hospital, Queensland; Queensland University of Technology (QUT), Australia; Jamieson Trauma Institute, Metro North Hospital and Health Service, Australia; ^4^The University of Queensland Faculty of Medicine, Australia

## Abstract

**Background:**

Infection following orthopaedic trauma surgery is increasingly recognized as one of the major research priorities with as primary goal, improving patient care. This increased interest has been anecdotally recognized through published research, research grants, and, finally, with the development of the fracture-related infection (FRI) consensus group. In 2017, the accepted consensus definition of FRI was published, which has been followed by consensus recommendations from both a surgical and medical perspective. A bibliometric analysis was performed to objectively describe the trends in published clinical research related to FRI.

**Methods:**

The terms related to FRI were searched in the Web of Science database between 2000 and 2020. The characteristics of clinical research on FRI regarding the author, country, journal, institution, scientific output, top 100 most cited articles, and trend topics were analyzed using Bibliometrix and WPS Office.

**Results:**

A total of 2597 records were eligible for inclusion in this bibliometric approach, with studies originating from 89 countries, including eight languages. The United States of America (USA) published the highest number of articles and citations. International collaborations were present between 72 countries, with the most active country being the USA. The most contributive institution was the University of California. The highest number of papers and citations were from the Injury-International Journal of the Care of the Injured and the Journal of Orthopaedic Trauma. The top 100 most cited articles were published in 27 different journals, with the number of citations ranging between 97 and 1004. The latest trend topics were related to the diagnosis of FRI.

**Conclusion:**

The present bibliometric analysis shows the research characteristics and trends of FRI from multiple perspectives. The fact that there is an increasing number of studies being published on FRI shows the agreement among scientists and clinicians that standardization with respect to this topic is very important.

## 1. Introduction

Internal fixation of fractures has revolutionized modern orthopaedic trauma care over the last 50 years. When indicated, internal fixation of fractures can preserve native joints and provide adequate stability to facilitate bone healing without limiting the functional rehabilitation of patients with prolonged immobilization [1]. However, postoperative infections complicate operative fracture management and occur in 1−11% of cases [2–6]. The diagnosis and treatment of postoperative infections are often complex. They require a multidisciplinary approach (i.e., surgeons, infectious disease physicians, and clinical pharmacists), often result in additional surgeries and a long duration of antibiotic therapy [7, 8]. The health care system costs are immense. Trauma patients with a postoperative infection generate inpatient costs 6.5-8 times higher than their uninfected counterparts [9]. Despite adequate treatment, clinical and functional outcomes often remain poor, with high dependency rates and an inability to return to work [10].

The terminology surrounding postoperative infections has been historically inconsistent, which has hampered research efforts. Terms such as infection after fracture fixation, surgical site infection, and septic nonunion (or pseudoarthrosis) were often used interchangeably but without clear criteria to define the entity [11, 12]. Therefore, in 2017, the term fracture-related infection (FRI) was defined by an international consensus group, including the AO Foundation, Orthopaedic Trauma Association, PRO IMPLANT Foundation, and European Bone and Joint Infection Society [13]. Over the course of the past decades, FRI has increasingly been recognized as a clinical research priority to improve the outcome of this sometimes devastating complication [14–16].

Bibliometrics is a method of statistical analysis used to assess a particular subject's characteristics and major developmental trends based on published research. Such an analysis has provided useful insight into the global state and trends related to different medical fields [17–21]. Anecdotally, there has been a significant increase in interest in the topic of FRI in recent years through published papers, targeted research grants, and consensus statements. However, till today, bibliometric analysis related to this topic has not been performed. The present study was aimed at describing the overview of clinical studies and trends on FRI between 2000 and 2020.

## 2. Materials and Methods

### 2.1. Data Sources and Search Strategy

Bibliometric analysis was performed using the electronic database Web of Science (Science Citation Index Expanded) for articles published between 2000 and 2020. The retrieval strategy used was for the following medical subject headings or keywords: “osteomyelitis,” “septic non-union,” “septic pseudoarthrosis,” “septic pseudarthrosis,” “infected non-union,” “infected pseudoarthrosis,” “infected pseudarthrosis,” “trauma∗,” “injur∗,” “fracture∗,” “fracture related infection,” “infection after fracture fixation,” “infections after fracture,” “infection after fracture osteosynthesis,” “post-traumatic osteitis,” “post-traumatic osteomyelitis,” “infections associated with fracture-fixation devices,” and “osteomyelitis associated with open fractures.”

### 2.2. Inclusion and Exclusion Criteria

Inclusion criteria for further analysis were based on the following: (a) article describing a clinical study on FRI in orthopaedics; and (b) review article, meta-analysis, clinical trial, or guideline.

Exclusion criteria were as follows: (a) book chapters, conference proceedings, editorials, errata, or letters; (b) basic science, animal, and cadaveric studies; and (c) nonorthopaedic related fracture or infection, such as of the skull or sternum.

### 2.3. Data Extraction and Bibliometric Analysis

Data were identified and extracted by three authors individually (CL, AF, NH). All records were exported from the Web of Science database with text and excel document then imported into the software of Bibliometrix (University of Naples Federico II, Italy) and WPS Office (Kingsoft, China) to analyze the result, respectively [22]. Data analysis involves the author, article title, country, journal, h-index, institution, author keywords, language, number of citations, number of publications, and publication year. The impact factor and quartile of the journal were collected from the Journal Citation Reports 2020. The Bibliometrix software was utilized to construct data visualization of international collaborations, top 10 authors' average output, and trend topics.

## 3. Results

### 3.1. Publication Output

**Figure 1:**
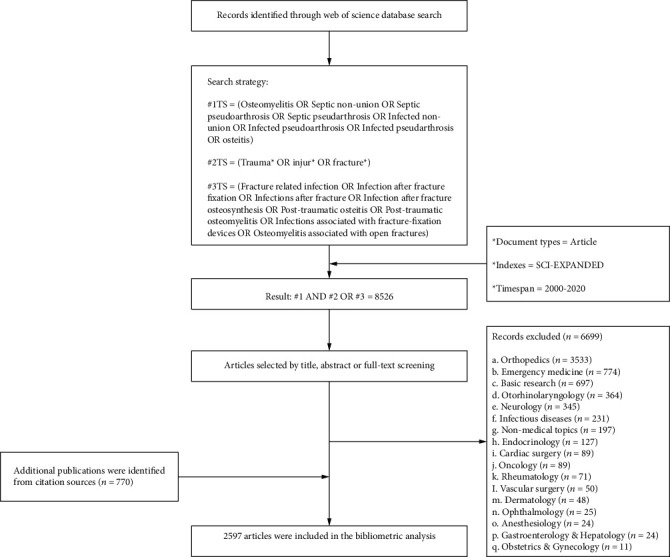
Flow chart of article selection.

A total of 2597 articles met the inclusion criteria using the electronic Web of Science database (Figure 1). The total number of citations related to FRI is 58,690 (51,109 excluding self-citations), with an average citation frequency of 22.6 times per item. Research papers were published in eight languages. More than 94% of articles were written in English (2458), followed by German (91), French (19), Czech (16), Serbian (4), Turkish (4), Spanish (4), and Italian (1).

**Figure 2:**
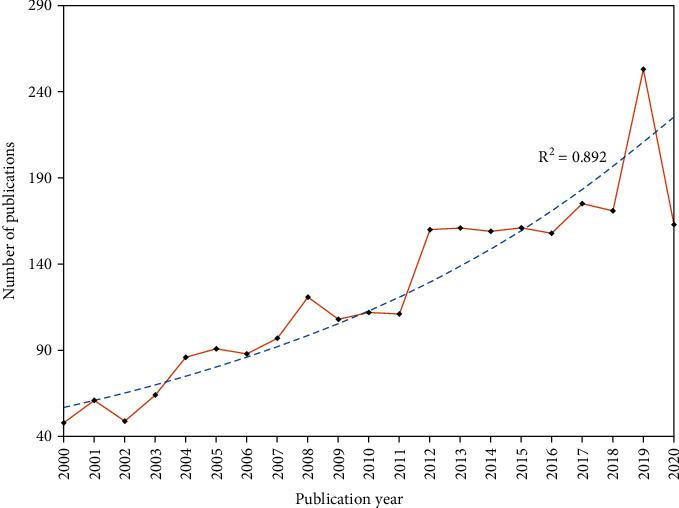
Number of publication outputs per year.

Figure 2 presents the specific amount of annual publications regarding FRI. Since 2008, the number of articles has exceeded 100 per year. The year 2019 ranked as the most productive year (253), followed by 2017 and 2018 (175 and 171, respectively).

### 3.2. Country

**Figure 3:**
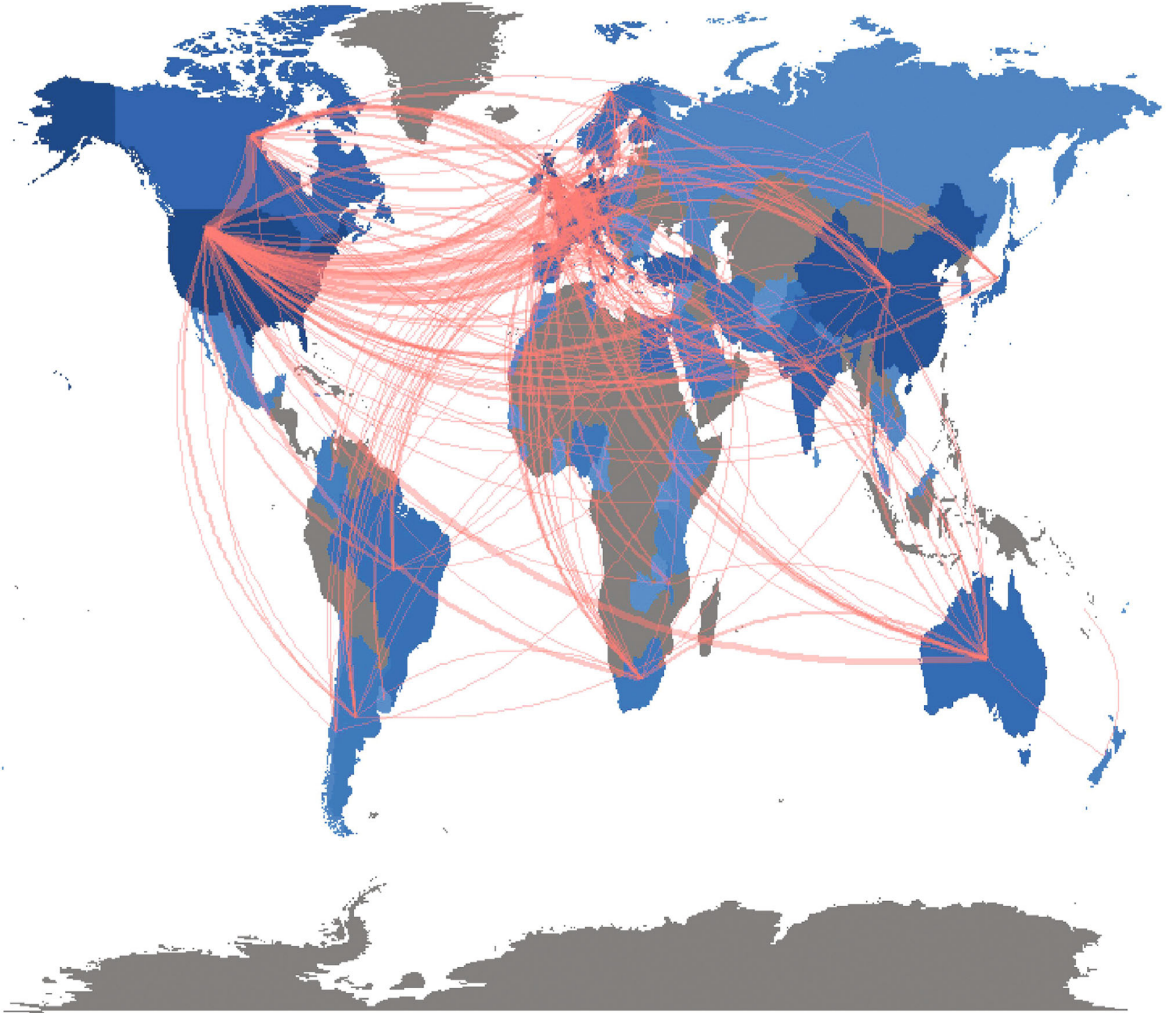
Global distribution and international interactions of FRI research.

**Figure 4:**
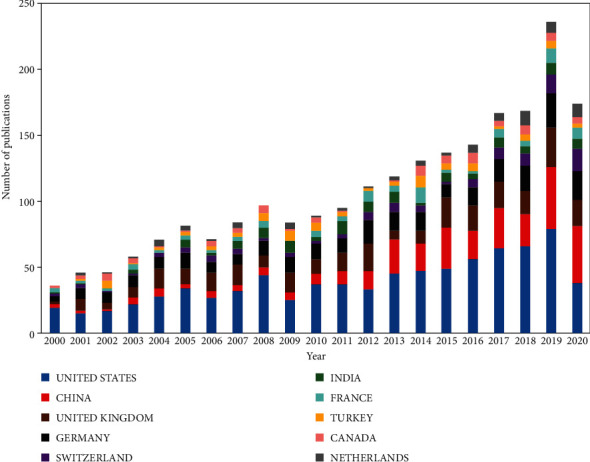
The annual distribution of FRI research from top 10 countries between 2000 and 2020.

For the United Kingdom (UK), publications from England, Northern Ireland, Scotland, and Wales were merged [23]. Finally, 89 countries contributed to the FRI publications (Figure 3). Of these, the USA contributed the highest number of articles (814), followed by China (318), the UK (298), and Germany (266; Table 1). The top four countries with the most contribution, each presented more than 10 articles per year between 2016 and 2020 (Figure 4).

Intercountry collaborations were found between 72 countries (Figure 3). The USA had the largest number of collaborations with other countries (46), followed by the UK (35) and France (31; Table 2). The most frequent collaborations were between the USA and Canada (40), followed by the USA and Germany (39), then Switzerland and Germany (32; Table 3).

### 3.3. Organizations

The top five most productive institutions are listed in Table 4. The most productive organization was the University of California system (48), followed by Harvard University (45) and Vanderbilt University (44). Of the top five most productive organizations, four were based in the USA, and the remaining was from the University Hospital Leuven, Belgium.

### 3.4. Authors

**Figure 5:**
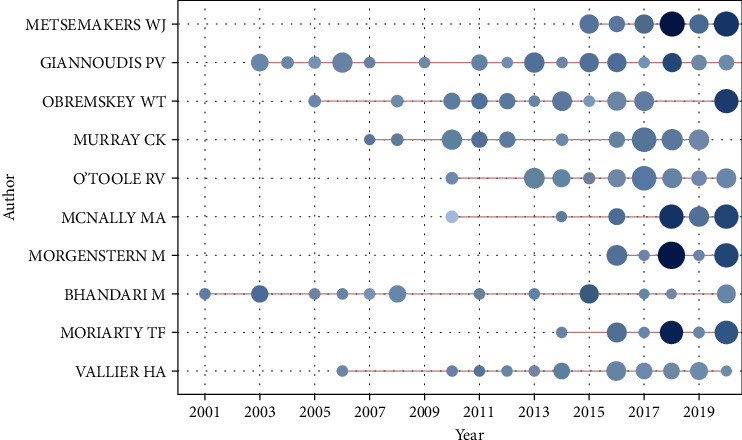
Average output of the top 10 authors on FRI-related studies between 2000 and 2020.

A total of 9,842 authors contributed to FRI-related studies. Metsemakers WJ has the highest number of publications with 32 articles, followed by Giannoudis PV (31) and Obremskey WT with 25 publications each (Table 5).  Figure 5 demonstrates the average output of the top 10 authors between 2000 and 2020.

### 3.5. Journals

**Figure 6:**
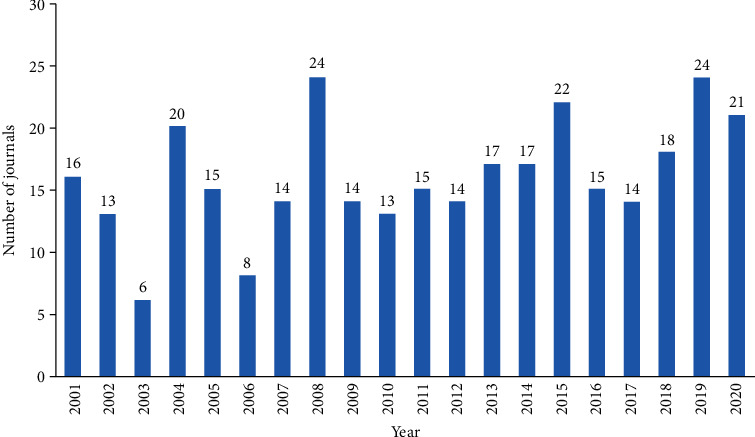
Year of first publication from journals on FRI.

All included articles were published in 339 different journals. Injury-International Journal of the Care of the Injured had the maximum number of papers (316), followed by the Journal of Orthopaedic Trauma (220) and International Orthopaedics (105; Table 6). The year of the first publication of an FRI study in the respective journal is depicted in Figure 6, with the majority appearing in 2008 and 2019 (24).

Data from the 2020 edition of journal citation reports showed 303 journals with an impact factor. The journal with the highest impact factor was The New England Journal of Medicine (74.699), followed by JAMA (Journal of the American Medical) Association (56.272), and Intensive Care Medicine (17.44; Table 7).

All of the top 10 journals with the most number of publications and impact factors were published in English. Journals with more than 10 publications in other languages included Unfallchirurg (German, 40), Revue de Chirurgie Orthopédique et Réparatrice de l'Appareil Moteur (French, 16), and Acta Chirurgiae Orthopaedicae et Traumatologiae Cechoslovaca (Czech, 16).

### 3.6. Top 100 Most Cited Articles

The number of citations for the top 100 most cited articles was defined according to the number of citations, with citations ranging between 97 and 1004 (Table 8). All included studies were published in 27 different journals, with most publications in the Journal of Orthopaedic Trauma (24), followed by Journal of Bone and Joint Surgery-American Volume (21), and Injury-International Journal of the Care of the Injured (9).

### 3.7. Trend Topics

**Figure 7:**
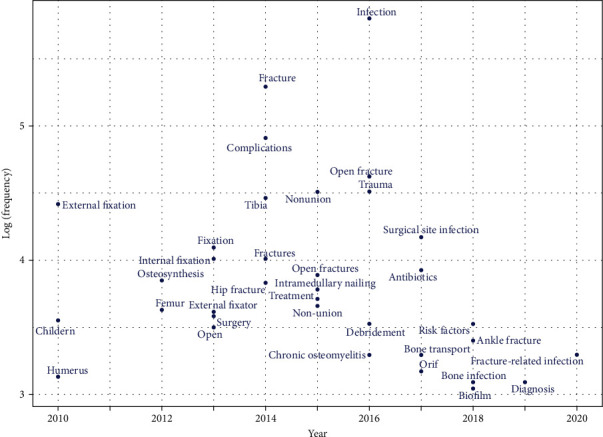
Trend topics of FRI according to author keywords.

Trend topics were identified by author keywords in Bibliometrix. Parameter values were set to a minimum number of keyword occurrences more than 20 times. The first keywords were presented until 2010, and 36 keywords met the threshold (Figure 7). The top three most frequently used keywords were “infection,” “fracture,” and “complications.” The earliest keywords were “external fixation,” “children,” and “humerus.” The keywords “fracture-related infection,” “diagnosis,” and “risk factor” were the top three latest emerging trend topics of FRI research.

## 4. Discussion

The current bibliometric study comprised a comprehensive analysis of the scientific output related to FRI, exploring characteristics from publications, languages, countries, institutions, authors, journals, most cited articles, and trend topics. In the current report, we provide scholars with essential references and suggestions for further investigation on FRI.

### 4.1. Global Publishing Trends

Although some research papers evaluated orthopaedic-related infection using bibliometrics [24, 25], to the best of our knowledge, this is the first article to fully assess clinical research on FRI. Our analysis shows that the number of related publications exhibited a significant increasing trend from 2000 to 2020. Of all languages, English was the main international scholarly language in this field. In addition, 65% of studies in languages other than English was in German. Several bibliometric analyses of medicine-related research also noted that German was the most common second language following English [19, 26].

### 4.2. Country

Half of the top 10 contributing countries on FRI studies originated from Europe, with the remaining coming from Asia and North America. The UK had the highest number of publications and academic influence in Europe, China in Asia, and the USA in North America. The annual distribution of FRI research from the top 10 countries displayed the USA ranked first in the top 10 largest contribution countries annually between 2000 and 2019. Interestingly, the UK and Germany persistently remained at around second and third place from 2003 to 2012 (Figure 4). China was ranked second between 2013 and 2019 and first in 2020. This result may suggest a great potential for the development of FRI research in China.

In 72 of 89 countries involved in international collaborations, the USA had the highest number of research collaborations with other countries. Most of the international collaborations were between North America and European countries. A greater international collaboration should be widely established in the future.

### 4.3. Organizations and Authors

Among the institutions, four of the top five highest contributing countries originated from the USA. The University of California from the USA was the most productive institution globally in clinical studies focusing on FRI. Scholars from Europe and North America demonstrated a dominant position. The publications on the diagnosis and treatment principles of FRI also proved that most members of the FRI consensus group originated from Euro-American countries [27, 28]. In addition, half of the top 10 most productive authors on FRI were involved in the FRI consensus group [13]. Metsemakers WJ, from the University Hospitals Leuven, contributed most to clinic research of FRI. Regarding the top 10 author productions over time, most authors showed increased stability and persistence for a longer duration between 2016 and 2020. The list of top 10 most productive authors in FRI may provide a valuable reference for future scientific conference invitations for FRI experts.

### 4.4. Journals

Of the top 10 journals with the largest number of papers, Injury-International Journal of the Care of the Injured had the highest number of relevant publications, whereas Journal of Orthopaedic Trauma was the most academic influential journal on FRI. Compared with the bibliometric study, which excludes non-English literature [29, 30], the present bibliometric research also provides more valuable journal information to non-English speaking countries. Hence, scholars will most likely benefit from journal information to further subscribe or track the most relevant journal and also submit clinical research of FRI manuscripts as a reference. Figures on the first publications indicated that an increasing number of journals are interested in FRI research, with the highest values attained in 2008 and 2019.

### 4.5. Most Cited Documents

The number of citations is a traditional indicator for the assessment of the value of a certain study. Our study displays the top 100 most impactful studies on FRI and provides a resource for clinical scientists. Of all publications, the most cited research paper was published by Govender et al. [31]. The authors found that an implant containing 1.50 mg/mL of recombinant human bone morphogenetic protein-2 in the treatment of open tibial fractures could reduce the incidence of postsurgical infections. The most cited review article was published by Trampuz and Zimmerli [32]. The authors summarized the pathogenesis, classification, diagnosis, and treatment of infections associated with fracture-fixation devices.

### 4.6. Trend Topics

Trend topics were identified from author keywords. In terms of the surgical site, scholars placed a greater interest in infection after humeral fractures, followed by the location of femur and tibia, with a current focus on ankle fractures [33–39]. Furthermore, research topics and concepts also altered over the course of the years. With the introduction of the FRI consensus group, the term “fracture-related infection” became the standard, with more recent topics primarily focusing on risk factors and diagnostic criteria of FRI [15, 40–43]. Prior to this, researchers focused on the treatment of FRI [44–46], with a focus on debridement, bone transfer, and antibiotic treatment. In addition, with the publication of the consensus definition of FRI, this concept appears to be gradually gaining ground in the scientific literature.

### 4.7. Limitations

There are several limitations to the present study. First, only a single database was searched, with other databases and sources not included in the bibliometric analysis. Therefore, some potentially valuable information may have been missed [47]. Second, the FRI-related terms of the search strategy were based on the literature and personal experience; nonetheless, some literature may have been overlooked. Third, conference proceedings were also removed from this bibliometric analysis, due to the potential of being published on two occasions, as a conference abstract and also as a full journal article [48, 49]. Fourth, the number of citations is commonly used to assess the publication quality in bibliometric analysis. To discover high academic impact publications in the clinical research of FRI, we listed the top 100 most cited articles. However, self-citation, publication date, and controversial articles all likely had an impact on the number of citations [21].

## 5. Conclusions

The number of articles on FRI showed an increasing trend over the last 21 years. English was the primary language used for academic exchange on the topic of FRI, followed by German. In addition, the USA has the most number and impactful publications, as well as international collaborations. China has great development potential in this field. A broader international collaboration on FRI is required in the future. The most relevant and academic influential journals on FRI are the Injury-International Journal of the Care of the Injured and the Journal of Orthopaedic Trauma, respectively. The definition of FRI will most likely become validated and widely available in clinical practice.

## Figures and Tables

**Table 1:** The top 10 most productive countries in FRI.

Countries	Records	Total citations	h-index
USA	814	25629	80
China	318	4256	34
UK	298	9383	49
Germany	266	6187	37
Switzerland	109	3139	31
India	107	1473	21
France	94	2708	21
Turkey	82	1081	19
Canada	81	5055	34
Netherlands	77	2854	27

**Table 2:** Top countries with more than 10 collaborations.

Ranks	Countries	Records
1	USA	46
2	United Kingdom	35
3	France	31
4	Germany	28
5	Canada	25
6	Switzerland	23
7	Netherlands	21
8	Australia	20
9	Belgium	20
10	Austria	18
11	South Africa	18
12	Israel	16
13	Norway	16
14	Argentina	15
15	China	15
16	Finland	15
17	Italy	14
18	Denmark	13
19	Greece	13
20	Ireland	12
21	Japan	11

**Table 3:** Countries with international collaborations exceeding fifteen.

From	To	Frequency
USA	Canada	40
USA	Germany	39
Switzerland	Germany	32
USA	UK	30
USA	Switzerland	27
Belgium	Switzerland	22
USA	Belgium	19
United Kingdom	Switzerland	18
USA	Netherlands	18
Belgium	Germany	17
UK	Belgium	16
UK	Germany	16

**Table 4:** The top five most prolific organizations in FRI.

Organization	Records	Total citations	h-index	Country
University of California System	48	2801	19	USA
Harvard University	45	1275	20	USA
Vanderbilt University	44	2097	22	USA
University System of Maryland	42	848	14	USA
University Hospital Leuven	37	1944	20	Belgium

**Table 5:** The top 10 most productive authors in FRI.

Author	Records	Total citations	h-index	Country	Affiliation
Metsemakers WJ	32	894	18	Belgium	University Hospitals Leuven
Giannoudis PV	31	1080	19	UK	University of Leeds
Obremskey WT	25	802	17	USA	Vanderbilt University
Murray CK	25	872	13	USA	San Antonio Military Medical Center
O'Toole RV	23	267	9	USA	University of Maryland
Mcnally MA	21	659	14	UK	Nuffield Orthopaedic Centre
Morgenstern M	20	635	13	Switzerland	University of Basel
Bhandari M	18	1231	14	Canada	McMaster University
Moriarty TF	17	586	13	Switzerland	AO Research Institute Davos
Vallier HA	17	502	9	USA	MetroHealth System

**Table 6:** Top 10 journals ranked by the number of publications on FRI.

Source	Number of publications	Total citations	h-index	Impact factor	Quartile in category
Injury-International Journal of the Care of the Injured	316	7352	44	2.586	Q2
Journal of Orthopaedic Trauma	220	8016	50	2.512	Q2
International Orthopaedics	105	1911	25	3.075	Q2
Journal of Trauma and Acute Care Surgery	91	3205	34	3.313	Q2
Bone & Joint Journal	85	3831	37	5.082	Q1
Archives of Orthopaedic and Trauma Surgery	76	1450	23	3.067	Q2
Journal of Bone and Joint Surgery-American Volume	72	6935	47	5.284	Q1
Clinical Orthopaedics and Related Research	68	2734	32	4.176	Q1
Journal of Pediatric Orthopaedics	63	1927	25	2.324	Q2
Foot & Ankle International	47	1304	20	2.827	Q2

**Table 7:** Top 10 journals ranked by the impact factor on FRI.

Source	Impact factor	Quartile in category	Number of publications	Total citations
New England Journal of Medicine	91.245	Q1	1	104
JAMA (Journal of the American Medical Association)	56.272	Q1	3	90
Intensive Care Medicine	17.440	Q1	1	23
British Journal of Sports Medicine	13.800	Q1	1	8
Bone Research	13.567	Q1	2	89
Biomaterials	12.479	Q1	1	260
Age and Ageing	10.668	Q1	1	54
Journal of Nuclear Medicine	10.057	Q1	1	131
European Journal of Nuclear Medicine and Molecular Imaging	9.236	Q1	6	172
Clinical Infectious Diseases	9.079	Q1	6	525

**Table 8:** The top 100 most cited articles on FRI.

First authors	Article title	Journals	Total citations	Year
Govender, S	Recombinant human bone morphogenetic protein-2 for treatment of open tibial fractures - a prospective, controlled, randomized study of four hundred and fifty patients	Journal of Bone and Joint Surgery-American Volume	1004	2002
Mckee, MD	Nonoperative treatment compared with plate fixation of displaced midshaft clavicular fractures - a multicenter, randomized clinical trial	Journal of Bone and Joint Surgery-American Volume	517	2007
Bhandari, M	Internal fixation compared with arthroplasty for displaced fractures of the femoral neck - a meta-analysis	Journal of Bone and Joint Surgery-American Volume	346	2003
Gopal, S	Fix and flap: the radical orthopaedic and plastic treatment of severe open fractures of the tibia	Bone & Joint Journal	332	2000
Trampuz, A	Diagnosis and treatment of infections associated with fracture-fixation devices	Injury-International Journal of the Care of the Injured	291	2006
Jamsen, E	Risk factors for infection after knee arthroplasty a register-based analysis of 43,149 cases	Journal of Bone and Joint Surgery-American Volume	277	2009
Flynn, JM	Titanium elastic nails for pediatric femur fractures: a multicenter study of early results with analysis of complications	Journal of Pediatric Orthopaedics	270	2001
Widmer, AF	New developments in diagnosis and treatment of infection in orthopedic implants	Clinical Infectious Diseases	263	2001
Rokkanen, PU	Bioabsorbable fixation in orthopaedic surgery and traumatology	Biomaterials	260	2000
Castillo, RC	Impact of smoking on fracture healing and risk of complications in limb-threatening open tibia fractures	Journal of Orthopaedic Trauma	239	2005
SooHoo, NF	Complication rates following open reduction and internal fixation of ankle fractures	Journal of Bone and Joint Surgery-American Volume	219	2009
Barei, DP	Complications associated with internal fixation of high-energy bicondylar tibial plateau fractures utilizing a two-incision technique	Journal of Orthopaedic Trauma	217	2004
Fankhauser, F	A new locking plate for unstable fractures of the proximal humerus	Clinical Orthopaedics and Related Research	212	2005
Pape, HC	Changes in the management of femoral shaft fractures in polytrauma patients: from early total care to damage control orthopedic surgery	Journal of Trauma-Injury Infection and Critical Care	210	2002
Yazar, S	One-stage reconstruction of composite bone and soft-tissue defects in traumatic lower extremities	Plastic and Reconstructive Surgery	205	2004
Hak, DJ	Delayed union and nonunions: epidemiology, clinical issues, and financial aspects	Injury-International Journal of the Care of the Injured	202	2014
Moro, JK	Arthroplasty with a metal radial head for unreconstructible fractures of the radial head	Journal of Bone and Joint Surgery-American Volume	201	2001
Stannard, JP	Negative pressure wound therapy after severe open fractures: a prospective randomized study	Journal of Orthopaedic Trauma	200	2009
Sproul, RC	A systematic review of locking plate fixation of proximal humerus fractures	Injury-International Journal of the Care of the Injured	197	2011
Schmidmaier, G	Prophylaxis and treatment of implant-related infections by antibiotic-coated implants: a review	Injury-International Journal of the Care of the Injured	194	2006
Bhandari, M	Treatment of open fractures of the shaft of the tibia - a systematic overview and meta-analysis	Bone & Joint Journal	188	2001
O'Neill, KR	Reduced surgical site infections in patients undergoing posterior spinal stabilization of traumatic injuries using vancomycin powder	Spine Journal	187	2011
Zalavras, CG	Local antibiotic therapy in the treatment of open fractures and osteomyelitis	Clinical Orthopaedics and Related Research	186	2004
Kregor, PJ	Treatment of distal femur fractures using the less invasive stabilization system - surgical experience and early clinical results in 103 fractures	Journal of Orthopaedic Trauma	185	2004
Johnson, EN	Infectious complications of open type III tibial fractures among combat casualties	Clinical Infectious Diseases	184	2007
Robinson, CM	Adult distal humeral metaphyseal fractures: epidemiology and results of treatment	Journal of Orthopaedic Trauma	177	2003
Koval, KJ	Fractures of the distal part of the radius	Journal of Bone and Joint Surgery-American Volume	174	2008
Yazar, S	Outcome comparison between free muscle and free fasciocutaneous flaps for reconstruction of distal third and ankle traumatic open tibial fractures	Plastic and Reconstructive Surgery	174	2006
Egol, KA	Staged management of high-energy proximal tibia fractures (OTA types 41) - the results of a prospective, standardized protocol	Journal of Orthopaedic Trauma	173	2005
Harris, AM	Complications following limb-threatening lower extremity trauma	Journal of Orthopaedic Trauma	169	2009
Nowotarski, PJ	Conversion of external fixation to intramedullary nailing for fractures of the shaft of the femur in multiply injured patients	Journal of Bone and Joint Surgery-American Volume	169	2000
Karger, C	Treatment of posttraumatic bone defects by the induced membrane technique	Orthopaedics & Traumatology-Surgery & Research	163	2012
Chapman, JR	Randomized prospective study of humeral shaft fracture fixation: intramedullary nails versus plates	Journal of Orthopaedic Trauma	162	2000
Arciola, CR	Etiology of implant orthopedic infections: a survey on 1027 clinical isolates	International Journal of Artificial Organs	161	2005
Vallier, HA	Randomized, prospective comparison of plate versus intramedullary nail fixation for distal tibia shaft fractures	Journal of Orthopaedic Trauma	158	2011
Im, GI	Distal metaphyseal fractures of tibia: a prospective randomized trial of closed reduction and intramedullary nail versus open reduction and plate and screws fixation	Journal of Trauma-Injury Infection and Critical Care	157	2005
Swiontkowski, MF	Recombinant human bone morphogenetic protein-2 in open tibial fractures - a subgroup analysis of data combined from two prospective randomized studies	Journal of Bone and Joint Surgery-American Volume	156	2006
Blauth, M	Surgical options for the treatment of severe tibial pilon fractures: a study of three techniques	Journal of Orthopaedic Trauma	155	2001
Stafford, PR	Reamer-irrigator-aspirator bone graft and bi Masquelet technique for segmental bone defect nonunions: a review of 25 cases	Injury-International Journal of the Care of the Injured	154	2010
Rechtine, GR	Postoperative wound infection after instrumentation of thoracic and lumbar fractures	Journal of Orthopaedic Trauma	151	2001
Minami, A	Vascularised fibular grafts - an experience of 102 patients	Bone & Joint Journal	150	2000
Barei, DP	Functional outcomes of severe bicondylar plateau fractures treated with dual incisions and medial and lateral plates	Journal of Bone and Joint Surgery-American Volume	148	2006
Prokuski, L	Prophylactic antibiotics in orthopaedic surgery	Journal of the American Academy of Orthopaedic Surgeons	146	2008
Nork, SE	Intramedullary nailing of distal metaphyseal tibial fractures	Journal of Bone and Joint Surgery-American Volume	145	2005
Skaggs, DL	Lateral-entry pin fixation in the management of supracondylar fractures in children	Journal of Bone and Joint Surgery-American Volume	145	2004
Pollak, AN	The relationship between time to surgical debridement and incidence of infection after open high-energy lower extremity trauma	Journal of Bone and Joint Surgery-American Volume	143	2010
Rodriguez-Merchan, EC	Nonunion: general principles and experimental data	Clinical Orthopaedics and Related Research	142	2004
Patzakis, MJ	Chronic posttraumatic osteomyelitis and infected nonunion of the tibia: current management concepts	Journal of the American Academy of Orthopaedic Surgeons	141	2005
McKee, MD	The use of an antibiotic-impregnated, osteoconductive, bioabsorbable bone substitute in the treatment of infected long bone defects: early results of a prospective trial	Journal of Orthopaedic Trauma	141	2002
Harley, BJ	The effect of time to definitive treatment on the rate of nonunion and infection in open fractures	Journal of Orthopaedic Trauma	140	2002
Ricci, WM	Locked plates combined with minimally invasive insertion technique for the treatment of periprosthetic supracondylar femur fractures above a total knee arthroplasty	Journal of Orthopaedic Trauma	139	2006
Naique, SB	Management of severe open tibial fractures - the need for combined orthopaedic and plastic surgical treatment in specialist centres	Bone & Joint Journal	138	2006
Mehlman, CT	The effect of surgical timing on the perioperative complications of treatment of supracondylar humeral fractures in children	Journal of Bone and Joint Surgery-American Volume	138	2001
Edwards, C	Early infection after hip fracture surgery - risk factors, costs and outcome	Bone & Joint Journal	137	2008
Knop, C	Late results of thoracolumbar fractures after posterior instrumentation and transpedicular bone grafting	Spine	137	2001
Sebastia-Forcada, E	Reverse shoulder arthroplasty versus hemiarthroplasty for acute proximal humeral fractures. A blinded, randomized, controlled, prospective study	Journal of Shoulder and Elbow Surgery	136	2014
Benirschke, SK	Wound healing complications in closed and open calcaneal fractures	Journal of Orthopaedic Trauma	132	2004
Filippi, L	Usefulness of hybrid SPECT/CT in Tc-99 m-HMPAO-labeled leukocyte scintigraphy for bone and joint infections	Journal of Nuclear Medicine	131	2006
Anglen, JO	Comparison of soap and antibiotic solutions for irrigation of lower-limb openfracture wounds - a prospective, randomized study	Journal of Bone and Joint Surgery-American Volume	131	2005
Leung, KS	Complex tibial fracture outcomes following treatment with low-intensity pulsed ultrasound	Ultrasound in Medicine and Biology	131	2004
Herrera, DA	Treatment of acute distal femur fractures above a total knee arthroplasty - systematic review of 415 cases (1981-2006)	Acta Orthopaedica	128	2008
Narayanan, UG	Complications of elastic stable intramedullary nail fixation of pediatric femoral fractures, and how to avoid them	Journal of Pediatric Orthopaedics	128	2004
Harvey, EJ	Morbidity associated with ORIF of intra-articular calcaneus fractures using a lateral approach	Foot & Ankle International	128	2001
Jost, B	Locking plate fixation of fractures of the proximal humerus: analysis of complications, revision strategies and outcome	Journal of Shoulder and Elbow Surgery	127	2013
Kettler, M	Treatment of proximal humeral fractures with the PHILOS angular stable plate. Presentation of 225 cases of dislocated fractures	Unfallchirurg	127	2006
Cassidy, C	Norian SRS cement compared with conventional fixation in distal radial fractures - a randomized study	Journal of Bone and Joint Surgery-American Volume	127	2003
Sirkin, M	A staged protocol for soft tissue management in the treatment of complex pilon fractures	Journal of Orthopaedic Trauma	124	2004
Metsemakers, WJ	Infection after fracture fixation: current surgical and microbiological concepts	Injury-International Journal of the Care of the Injured	121	2018
Stulik, J	Minimally-invasive treatment of intra-articular fractures of the calcaneum	Bone & Joint Journal	119	2006
Khatod, M	Outcomes in open tibia fractures: relationship between delay in treatment and infection	Journal of Trauma-Injury Infection and Critical Care	119	2003
Cole, PA	Treatment of proximal tibia fractures using the less invasive stabilization system - surgical experience and early clinical results in 77 fractures	Journal of Orthopaedic Trauma	117	2004
Springer, BD	Treatment of periprosthetic femoral fractures following total hip arthroplasty with femoral component revision	Journal of Bone and Joint Surgery-American Volume	117	2003
Metsemakers, WJ	Fracture-related infection: a consensus on definition from an international expert group	Injury-International Journal of the Care of the Injured	116	2018
Gosling, T	Single lateral locked screw plating of bicondylar tibial plateau fractures	Clinical Orthopaedics and Related Research	116	2005
Takeuchi, R	Fractures around the lateral cortical hinge after a medial opening-wedge high tibial osteotomy: a new classification of lateral hinge fracture	Arthroscopy-the Journal of Arthroscopic and Related Surgery	115	2012
Canadian Orthopaedic Trauma Society	Open reduction and internal fixation compared with circular fixator application for bicondylar tibial plateau fractures - results of a multicenter, prospective, randomized clinical trial	Journal of Bone and Joint Surgery-American Volume	113	2006
Yun, HC	Osteomyelitis in military personnel wounded in Iraq and Afghanistan	Journal of Trauma-Injury Infection and Critical Care	111	2008
Taeger, G	Damage control orthopedics in patients with multiple injuries is effective, time saving, and safe	Journal of Trauma-Injury Infection and Critical Care	111	2005
Parsons, B	Surgical management of chronic osteomyelitis	American Journal of Surgery	111	2004
Lack, WD	Type III open tibia fractures: immediate antibiotic prophylaxis minimizes infection	Journal of Orthopaedic Trauma	109	2015
Borg, T	Percutaneous plating of distal tibial fractures preliminary results in 21 patients	Injury-International Journal of the Care of the Injured	108	2004
Parameswaran, AD	Pin tract infection with contemporary external fixation: how much of a problem?	Journal of Orthopaedic Trauma	108	2003
Suk, SI	Anterior-posterior surgery versus posterior closing wedge osteotomy in posttraumatic kyphosis with neurologic compromised osteoporotic fracture	Spine	106	2003
Misra, A	Complex proximal humeral fractures in adults - a systematic review of management	Injury-International Journal of the Care of the Injured	106	2001
Bhandari, M	A trial of wound irrigation in the initial management of open fracture wounds	New England Journal of Medicine	104	2015
Aro, HT	Recombinant human bone morphogenetic protein-2: a randomized trial in open tibial fractures treated with reamed nail fixation	Journal of Bone and Joint Surgery-American Volume	104	2011
Ferran, NA	Locked intramedullary fixation vs plating for displaced and shortened mid-shaft clavicle fractures: a randomized clinical trial	Journal of Shoulder and Elbow Surgery	104	2010
Rozbruch, SR	Simultaneous treatment of tibial bone and soft-tissue defects with the ilizarov method	Journal of Orthopaedic Trauma	104	2006
Karunakar, MA	Body mass index as a predictor of complications after operative treatment of acetabular fractures	Journal of Bone and Joint Surgery-American Volume	104	2005
Feldman, DS	Correction of tibial malunion and nonunion with six-axis analysis deformity correction using the Taylor spatial frame	Journal of Orthopaedic Trauma	104	2003
Edlund, A	Delirium before and after operation for femoral neck fracture	Journal of the American Geriatrics Society	104	2001
Richards, JE	Relationship of hyperglycemia and surgical-site infection in orthopaedic surgery	Journal of Bone and Joint Surgery-American Volume	102	2012
Gjertsen, JE	More re-operations after uncemented than cemented hemiarthroplasty used in the treatment of displaced fractures of the femoral neck. An observational study of 11 116 hemiarthroplasties from a national register	Bone & Joint Journal	101	2012
Nasell, H	The impact of smoking on complications after operatively treated ankle fractures-a follow-Up study of 906 patients	Journal of Orthopaedic Trauma	100	2011
Lau, TW	Wound complication of minimally invasive plate osteosynthesis in distal tibia fractures	International Orthopaedics	100	2008
Fuchs, T	The use of gentamicin-coated nails in the tibia: preliminary results of a prospective study	Archives of Orthopaedic and Trauma Surgery	99	2011
Flynn, JM	Management of pediatric femoral shaft fractures	Journal of the American Academy of Orthopaedic Surgeons	99	2004
Malik, MHA	Factors affecting rates of infection and nonunion in intramedullary nailing	Bone & Joint Journal	99	2004
Collinge, C	Anterior-inferior plate fixation of middle-third fractures and nonunions of the clavicle	Journal of Orthopaedic Trauma	98	2006
Mckee, MD	A prospective, randomized clinical trial comparing an antibiotic-impregnated bioabsorbable bone substitute with standard antibiotic-impregnated cement beads in the treatment of chronic osteomyelitis and infected nonunion	Journal of Orthopaedic Trauma	97	2010

## Data Availability

Data is available from the first author upon request.

## Abbreviations

FRI: Fracture-related infection
USA: United States of America
UK: United Kingdom.
